# Open data for energy networks: introducing DAVE—a data fusion tool for automated network generation

**DOI:** 10.1038/s41598-024-52199-w

**Published:** 2024-01-22

**Authors:** Tobias Banze, Tanja M. Kneiske

**Affiliations:** 1 Fraunhofer Institute for Energy Economics and Energy System Technology (IEE), 34119 Kassel, Germany; 2 Fraunhofer Institute for Energy Infrastructures and Geothermal Systems (IEG), 03046 Cottbus, Germany

**Keywords:** Energy infrastructure, Energy grids and networks, Power distribution, Power stations

## Abstract

Developing a sustainable energy system for the future requires new ways of planning and operating energy infrastructure. A large part of this involves suitable network models. Real network data is not available for research without restrictions since energy networks are part of the critical infrastructure. Using open datasets and expert rules to generate non-restricted models is one solution to this. This paper introduces open data for energy networks generated by the open-source software “DAVE”. The Python-based data fusion tool DAVE can automatically generate customized energy network models quickly and on demand. The software collects data from various databases and uses appropriate methods to fuse them. The current version of the tool can create GIS-based power networks and gas transportation networks, with output that is compatible with common network simulation software. Further developments are planned for creating thermal and gas distribution networks, as these are important for local heat power transition. Implementing a quality description for fused datasets will also be included in future development.

## Introduction

An important and necessary part of the simulation and analysis of energy infrastructures are appropriate network models. Currently, it is common practice to manually prepare network data to generate these models. This takes a lot of time and knowledge. In order to reduce this demand on resources, the modeling of energy networks needs to become faster and more efficient. This can be achieved by automating the network modeling process. The most accurate models are generated using real network data which only the system operators have. Since energy networks are part of the critical infrastructure these data are not available for research without restrictions like only use as part of a cooperation with the system operator. This often means that results based on confidential data cannot be published in detail, which affects the validation of studies by independent researchers. For research it is important that the network models are open to use, even if the trade-off is that the data is less accurate. This provides the opportunity to publish the results of studies and to make them comprehensible as well as comparable. The use of open data can achieve this.

**Figure 1:**
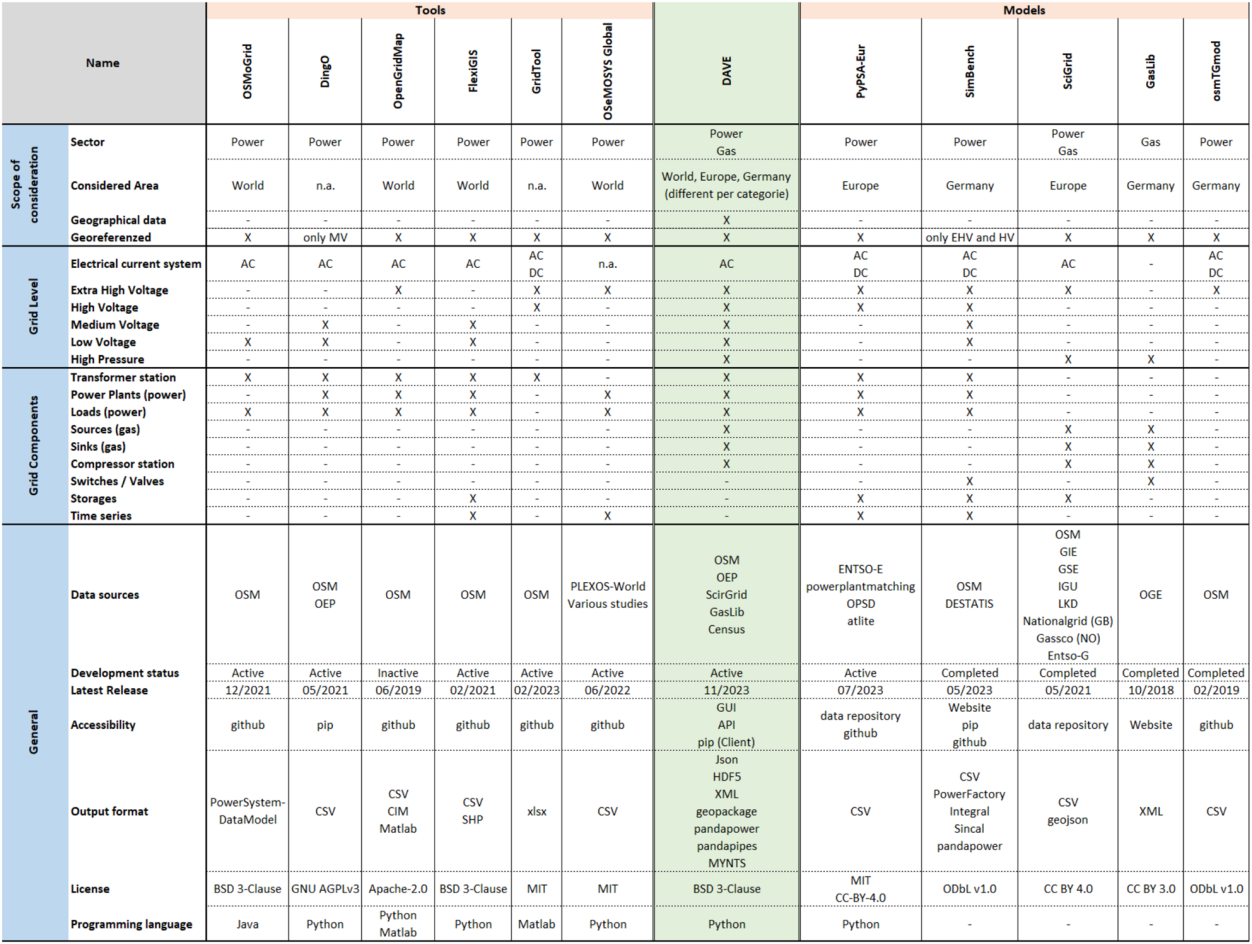
Overview about existing tools and models in comparison with DAVE. Tools: OSMoGrid^7,8^, Ding0^9,10^, OpenGridMap^11^, FlexiGIS^12^, GridTool^13^ and OSeMOsys Global^14^. Models: PyPSA-Eur^15,16^, SimBench^17,18^, SciGRID^19–21^, GasLib^22^ and osmTGmod^23^.

There are some software tools and network models that add value to the development of energy network models. While these mostly focus on specific voltage or pressure levels with a high level of detail, DAVE follows another approach. The open-source software tool introduced in this paper will cover a wide range of energy network data. This will provide the opportunity to only have to use this one tool disregarding for which network level or components a model is required. In addition, this provides the opportunity to create cross-sectoral coupling models. Figure 1 presents an overview of the characteristics of the mentioned tools and models in comparison with DAVE. It also shows the type of data covered by DAVE.

The development of DAVE started in 2020 as part of the “eGo$^n$”^1^ project. Here, the basic framework with a focus on electricity network models was developed in the context of a master thesis^2^. Subsequently, the extension of the tool was developed as part of a number of other government-funded projects. The existing network model generation has been tested and improved during the work on the “GridCast”^3^ and “Inteever II”^4^ projects. A software architecture and development structure were designed within the “H2D” project. This makes it easier to collaborate with other research institutions to bundle the knowledge of experts in different fields. The ability to generate gas transport network models has also been added. At present, the provision of geodata relevant to network expansion planning, for example, is being expanded in the “ANaPlan Plus”^5^ project. Another ongoing project, “TransHyDE”^6^, is improving the gas sector and adding information on potential hydrogen networks. DAVE is constantly evolving and there are many possible extensions to the range of considerations, such as gas distribution networks, heat networks and improving data quality for use in complex calculations.

**Figure 2:**
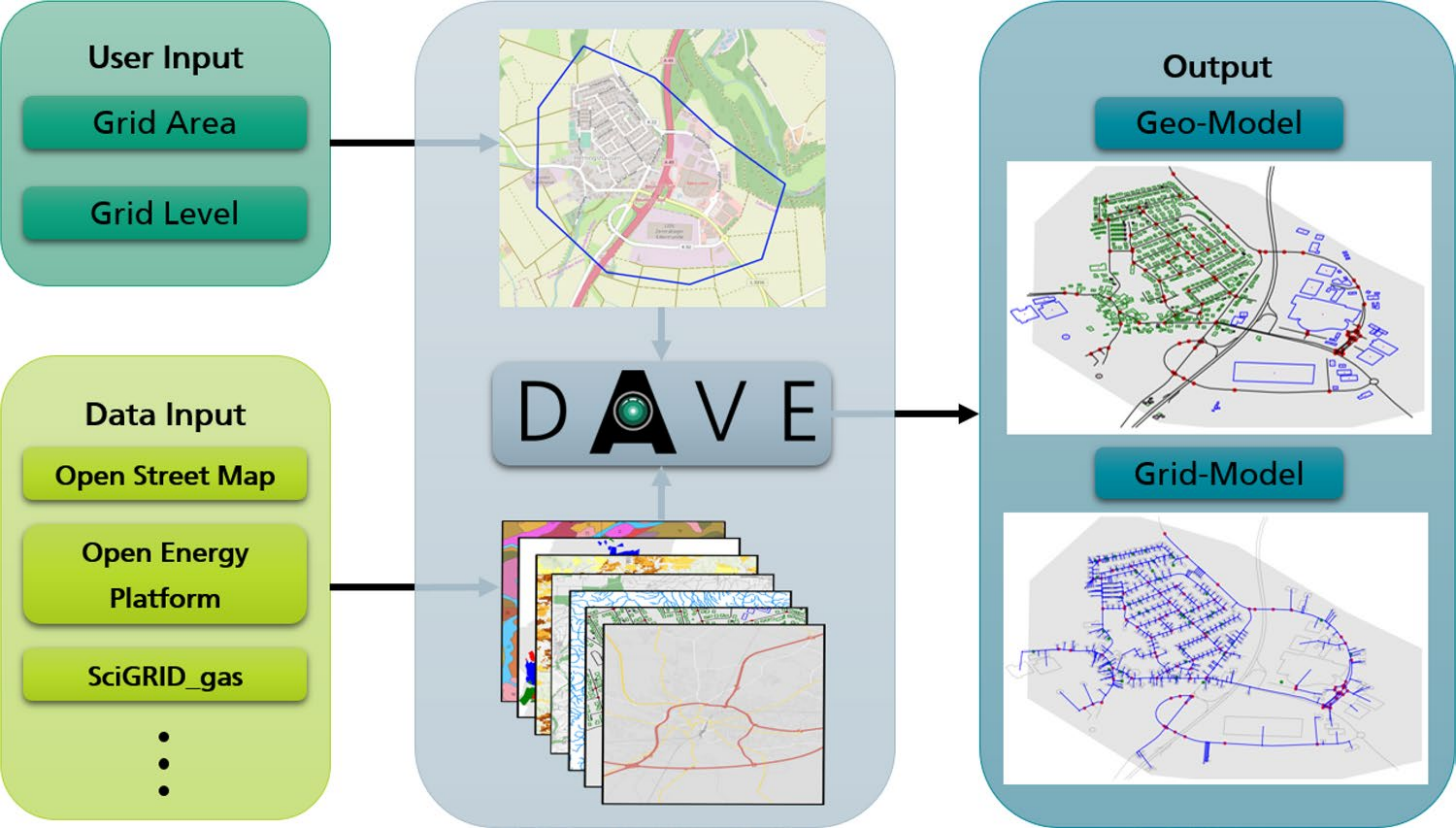
Schematic approach of the data fusion tool DAVE.

DAVE was developed to automatically create network models from available open datasets and provide them in easy-to-use data formats of commercial and open-source software commonly used for network simulations^2^. It bridges the gap between georeferenced data analysis and infrastructure simulation. Following this idea, DAVE does publish some new network-related datasets, but mainly accesses open-data databases to obtain required information, fuse it, fill the gaps, and format it into structures of established network simulation software. Furthermore, it covers a wide range of potential user requirements, allowing high flexibility in the choice of the network model to be created. DAVE, with the possibility to provide the combined data in raw format, is intended to appeal to both professional users and users who are new to power systems by offering the possibility to provide some basic network model editing and optimization functions to obtain computable network models. To achieve very high usability, DAVE requires minimal input from the user and assumes no knowledge of GIS data, network model data formats or network modeling. For a general overview, the schematic approach of the tool is shown in Fig. 2.

## Methods

**Figure 3:**
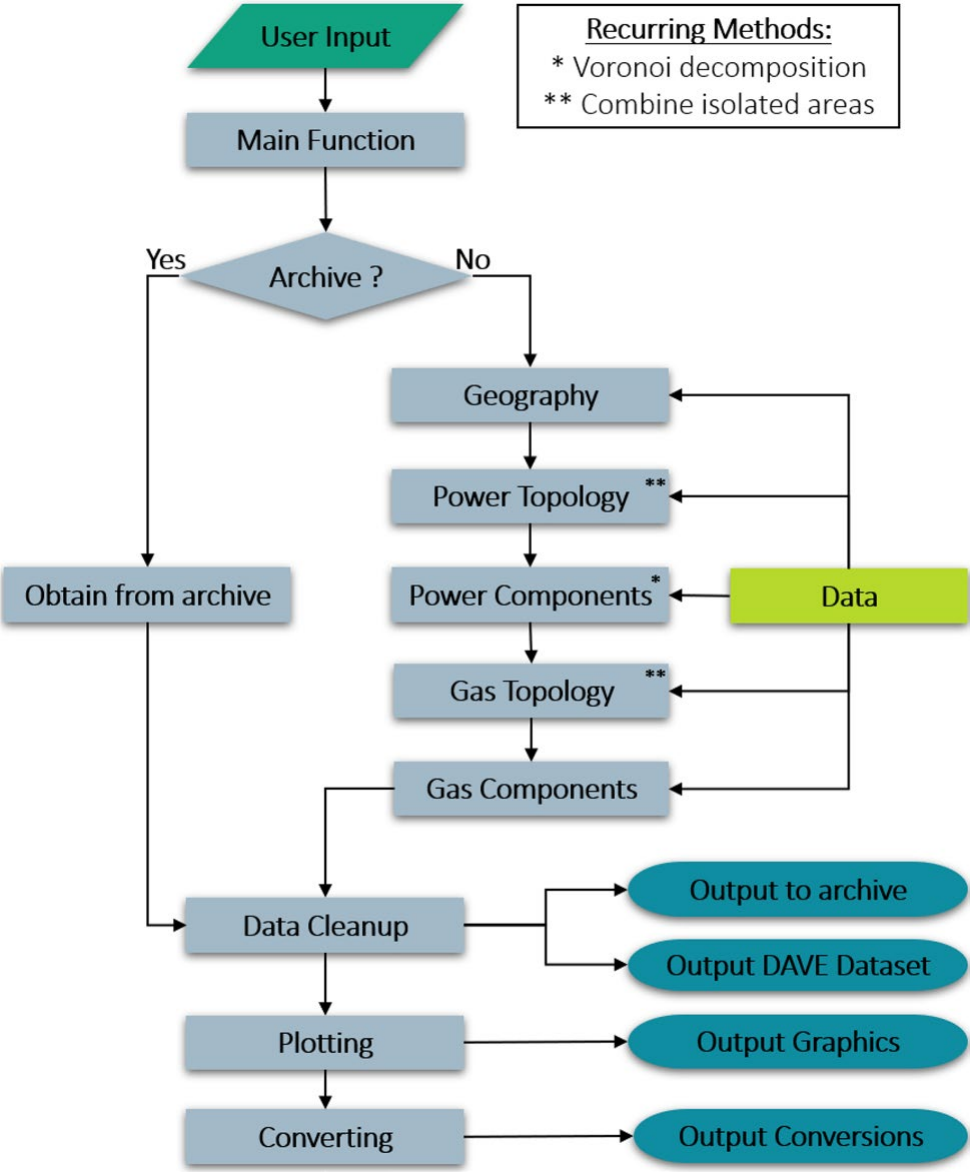
Flowchart of the DAVE core process. The colors are matching to Fig. 2.

The following subsections present the automatic network model generation of the DAVE tool. These subsections correspond to the order of the generation process shown in Fig. 3. Firstly, there will be a description of what input information is required from the user and what data sources are in use. This information forms the basis for the network model generation process, which is presented in detail below. Finally, some information about the data output and the outlook on DAVE is provided.

### User input

The user specifies the desired voltage- and/or pressure-levels and the area of interest. Currently, all electricity network levels and the high-pressure gas level can be selected. To define the network area, the user has the possibility to use postal codes (Germany), town names (Germany), federal state names (Germany), NUTS regions (Europe, independent of the NUTS level) or to submit the desired area as an polygon object. Under these conditions, it is not necessary for the user to have prior knowledge of energy network modeling, data processing or programming in Python to use DAVE.

### Data

The data processed in DAVE is collected from various available open data sources and stored in a dedicated database to reduce runtime during the generation process. Some of the source data can be accessed by the tool through an application programming interface (API). Data sources that currently provide the most relevant information and also have a user interface are “OpenStreetMap” (OSM)^24^ and “Open Energy Platform” (OEP)^25^. The function to query data from OSM is mainly based on the Python package “geopandas_osm”^26^, which is available under the MIT license. The code was implemented in DAVE and adapted to work with other packages used. By using this feature, there is the ability to obtain information about each tag for a defined area from OSM. To query data from the OEP database, a script was developed that uses the OEP built-in API. Within this function, there is the ability to query any schemas and tables from the OEP with a data filter. In addition, the function converts geographic information into a suitable geometric Python object. In case the data source has an API, functions are implemented for the DAVE database to periodically check for updates automatically and modify the data if necessary. In addition, retrieving the data via the API serves as a backup in case the DAVE database is unreachable. However, not every data source has a database with a suitable API to retrieve its dataset. In this case, the information is downloaded without a regular routine and stored in raw format in DAVE’s own database which is located on a server at the Society of scientific data processing GmbH Göttingen (GWDG)^27^. Within DAVE, there are appropriate read-in functions for each dataset that also prepare the data for the next steps in the process. In addition, each element is given a parameter that names the original source. The metadata for each dataset used is also stored in the resulting DAVE dataset. A detailed description of what kind of data from which source is currently used is presented later in the “Datasets” section. In addition, to improve performance, a separate archive system has been built. The associated function stores the results of a queried dataset so that it can be used when a dataset is queried again with the same settings. The adjustment necessary for this is managed via an inventory file. In this case, DAVE saves runtime by reusing the existing results instead of going through the entire generation procedure.

### DAVE process

#### Recurring methods

Before we look in more detail at the model generating process of DAVE, two methods are described that recur in different parts of the process. The parts where they are used can be seen in Fig. 3.

##### Voronoi decomposition

 Voronoi decomposition is a method of subdividing space, based on some points called centroids. In the case of energy network modeling, such points could be for example the nodes or substations of a power network. The approach of Voronoi analysis is based on the idea of assigning to each point in space a centroid area by searching the centroid which has the shortest distance to them. For this decision, the method uses the Euclidean distance. A corresponding algorithm has been integrated into DAVE. In addition to the basic algorithm, this one computes the extreme points by creating a convex hull for the centroids and taking the edge points from it. These points are then added to the set of centroids that results from the Voronoi decomposition by restricting the defined regions for the centroids in question so that they become finite. This step is important to obtain closed polygons for further geometric considerations. This method can be used, for example, to allocate energy production or demand to substations in the context of power network modeling.

##### Combine isolated areas

**Figure 4:**
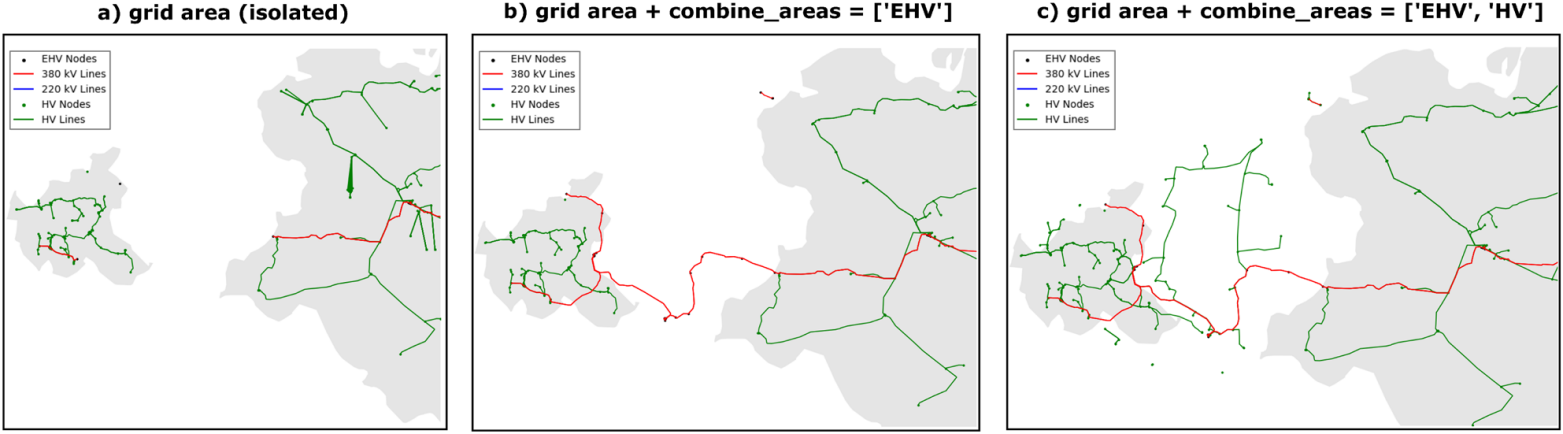
Scheme for the combine areas method in DAVE.

 There is an optional function in DAVE to connect areas that are isolated from each other. An example for such a case is presented in Fig. 4a. The user can select the network layers to be used for this connection via the “Combine Areas” parameter in the main function. In order to minimize the impact of the additional area used to connect isolated areas, it is only considered during the creation of the network topology. Therefore, the first step in connecting isolated areas is to cache the original user defined network area before the topology processes begin. Then, the network area definition is extended to include additional regions that fill the gaps in between. To do this, the isolated areas are first identified by iterating over all the given areas and checking whether they have a minimum distance greater than zero from the other areas. A pair of regions consisting of the isolated region and the region with the minimum distance to it is defined if such a case is found. Afterwards, iteration is performed over the defined area pairs, merging the two areas into a polygon and computing its convex hull. Then the difference between the convex hull and the original areas is determined. This results in a polygon that represents the gap, between the considered area and is added to the network area information. The additional area definition is labelled “intermediate area” in DAVE. Then, the topology processes described earlier are run and both the interim area and the user-entered area are considered for the network levels defined in the “Combine Areas” parameter. The described process is visualized in Fig. 4b for one network level selected for the combining isolated areas and in Fig. 4c for multiple levels. If the user additionally queries network levels that are not to be used for combining isolated areas, only the definition of the original user defined area for this levels is used. After the topology processes are finished, the network area definition is reset to the original area for further generation steps.

#### Main function

Based on DAVE’s philosophy to keep the creation of an individual energy network dataset simple for the user, there is only one main function that performs the entire process (see Fig. 3). With the parameters that the user sets in this function, DAVE internally defines the flow of the process by automatically performing the necessary tasks. DAVE first creates an empty attribute dictionary with its own structure and prepares the user inputs to be suitable for further steps. For example, the main function puts the network-level inputs in the correct order and corrects for case sensitivity. In each of the subsequent steps of the process, the DAVE dictionary is expanded to include the collected and adjusted information. The generated network data is given its own naming structure. The names are composed of the type of network element, the network level, and a sequence number. In addition, the network level for each element is added as a separate parameter. Basically, the coordinate system “EPSG:4326” is used for geographic data. This coordinate system is based on longitude and latitude and is available for the entire world. For distance calculations, a projection is made internally to the “EPSG:3035” coordinate system. This system provides more accurate results because it has been geocentrically translated into a two-dimensional view. It has meters as its unit and is available for the region of Europe. After the data fusion is complete, the resulting dataset is stored in an internal archive. DAVE can fall back on this archive if another query with the same features is made, which shortens the runtime. The more important part is that the resulting dataset is returned to the user. For this, the user has the possibility to define the output format. For a better overview of how the individual process steps are linked, Fig. 3 shows the core of DAVE in a flowchart. In addition, each generation step is described in detail in the following subchapters.

#### Geography

Based on the user input, an algorithm creates the area of interest by reading and filtering the area information from the database. The resulting geographic description of the area is written to the DAVE dataset and is important because it is used in the subsequent steps to reduce the data to the area of interest. Also, some geographic information for the area is collected and added as well. This information can currently include roads, buildings, land use areas, railways and waterways based on OSM as data source. The roads are used to generate network topologies and for better orientation in plotting. For building data, DAVE distinguishes between commercial and residential, which is used, for example, in the derivation of low-voltage networks. Industrial buildings belong to the commercial category. The land use data describes how an area is used. For a complete dataset the three different OSM keys landuse, leisure and natural will be consider. This information is useful, for example, when calculating loads in power networks. For a consideration with lower spatial resolution respectively for higher network levels, this load generation approach is more sensible and faster than a consideration of the single buildings. In this case it is depending on the usage because e.g. a residential area has another power consumption than an industrial area. In addition, the land use data is used to correct the building information from OSM for the buildings marked “yes”. To obtain this information, the paths and relations from OSM are queried for the corresponding tag. The geometry information of the resulting data is then converted into geometric objects and the area is calculated. Knowledge of railroads and waterways in the area under consideration can be helpful in network extension considerations. DAVE uses the input from the user for the network layers as well as the network components to decide which geographical data are necessary for the next steps and collects them. The data comes from the OpenStreetMap platform^24^ and is automatically queried using the API described earlier in “Data” section. The query only retrieves data for OSM tags predefined in tool. The Table 1 shows an overview of the tag keys and values used. In addition, DAVE derives road intersections from the road information for later use in linking low voltage power lines. The development plan is to expand the coverage of geographic information (e.g., soil texture or fertility, protected areas, etc.) as this data is relevant to network expansion planning.

**Table 1:** OpenStreetMap tags are used in DAVE to collect geographical data.

Type	Road	Building residential	Building commercial	Landuse	Railway	Waterway
Keys	Highway	Building	Building	Landuse Leisure Natural	Railway	Waterway
Value	Secondary	Apartments	Commercial	Landuse: true	Construction	River
	Tertiary	Detached	Hall	Leisure: golf_course	Disused	Stream
	Unclassified	Dormitory	Industrial	Leisure: garden	Light_rail	Canal
	Residential	Dwelling_house	Kindergarten	Leisure: park	Monorail	Tidal_channel
	Living_street	Farm	Kiosk	Natural: scrub	Narrow_gauge	Pressurised
	Footway	House	Office	Natural: grassland	Rail	Drain
	Track	Houseboat	Retail	Natural: water	Subway	
	Path	Residential	School	Natural: wood	Tram	
	Motorway	Semidetached_house	Supermarket			
	Trunk	Static_caravan	Warehouse			
	Primary	Terrace				
		Yes				

#### Power topology

**Figure 5:**
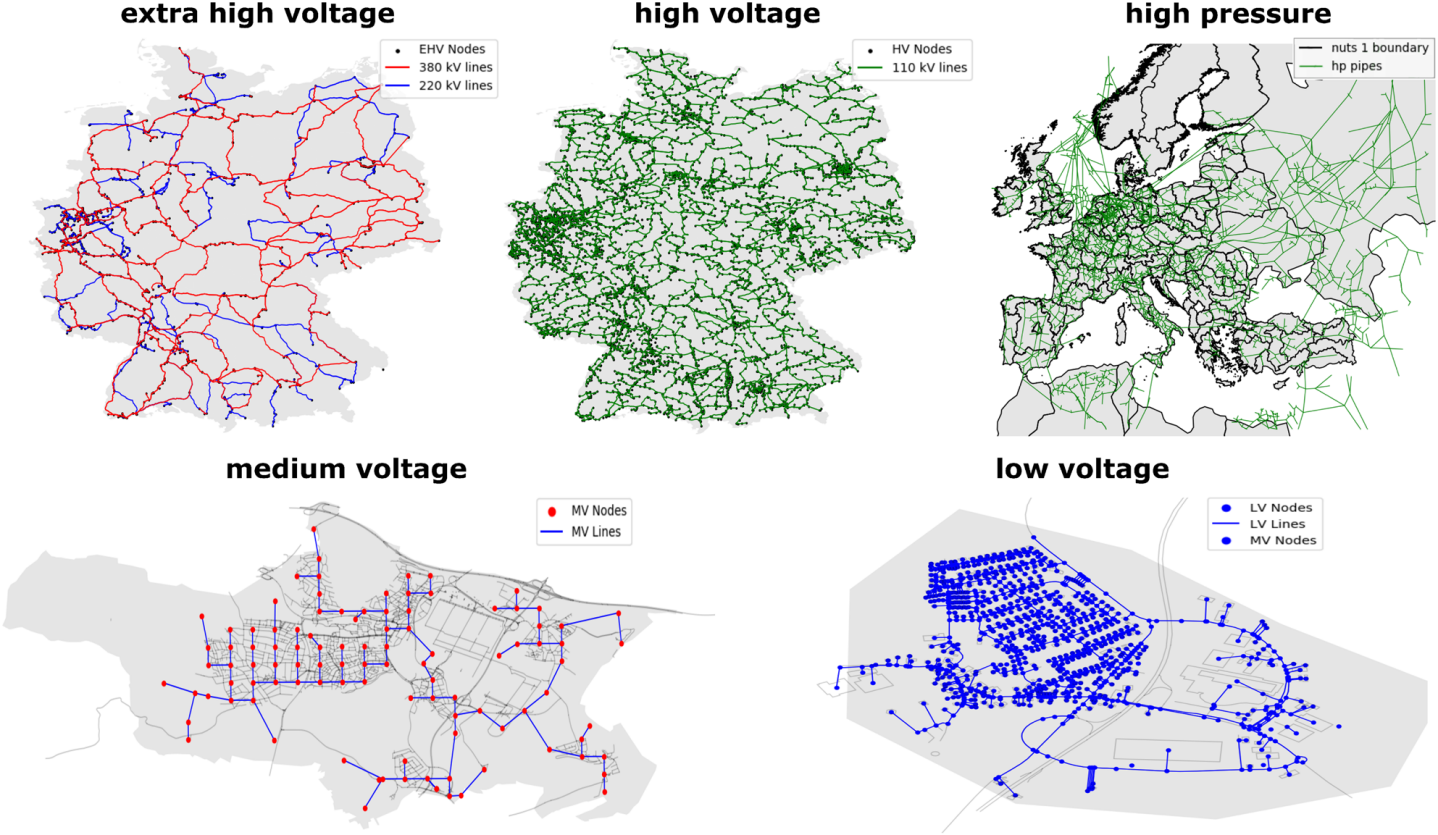
Example of resulting network topology for each level.

The next step in the DAVE process chain is to aggregate and calculate the network topology data for the power sector. Topology in this context includes georeferenced nodes and lines. It is currently possible to generate data for all voltage network levels (extra-high, high, medium, and low voltage). Due to the data situation on the different network levels, different methods are required, which are described in more detail below. In general, the higher the voltage level, the better the quality of available open data because higher voltage network overhead line geometries are registered in OSM, as opposed to underground cables in low voltage networks. This leads to more complex and predictive approaches at medium and low voltage levels. Figure 5 shows an example of a resulting network topology for each level.

##### Extra high and high voltage level

 When creating the extra-high voltage (ehv) and high voltage (hv) topology, first the information for the substations (datasets: “ego_dp_ehv_substation”^28^ and “ego_dp_hvmv_substation”^29^), nodes (data set: “ego_pf_hv_bus”^30^) and lines (dataset: “ego_pf_hv_line”^31^) will be requested from the Open Energy Platform via the REST-API. The related functions for the extra high and high voltage levels are independent of each other within the data generation process of DAVE, but because of the same approach to generating the two levels, they are described together in this section. Due to the initial data situation, the generation is currently limited to the area of Germany and the AC networks. After the data for all three element types (substations, nodes, lines) are available, filtering is performed to select the elements that are in operation at the current time, have the correct voltage level, and are located in the area under consideration. For the line elements, in addition to the intersection with the considered area, the rule is that at least one of the start or end point of the line must be within the area. This rule also implements lines in the resulting DAVE dataset that connect surrounding network areas and are necessary for the power exchange of the network. This additionally leads to the inclusion of some start or end points that are located outside the considered area. For better linkage of the information in the DAVE dataset itself, an algorithm checks for each node whether it is located in a substation area or in its immediate vicinity, and assigns the substation name in case.

##### Medium voltage level

 The creation of the medium voltage (mv) topology is mostly based on assumptions, as there is almost no public data for this voltage level. The reason for this is the underground installation, which is documented confidentially by network operators and thus not publically available. DAVE follows an approach where the nodes are taken from the substation locations. First, the substation information (dataset: “ego_dp_hvmv_substation”^29^ and “ego_dp_mvlv_substation”^32^) is requested from the Open Energy Platform. A network node is then generated for each substation location that lies within the user-defined network area. Since this substation information is used for the network nodes, the generation of the medium voltage topology is currently limited to Germany. Based on the generated nodes, the network lines are calculated, where each node is connected to the other with a minimum distance. To fulfil this condition, the first part of the method iterates through the nodes and finds the closest matching node. The pair of nodes found is removed from the set of possible nodes for the further iteration steps. After all, nodes have their partner and a line is created to connect them. The second part of the method iterates over the line segments and their connection to the nearest neighbour line until all line segments are connected to each other. In the process, larger and larger line segments are formed, eventually creating a graph that connects all the nodes.

To make these resulting topology more realistic, the lines should follow the course of the roads. At present there is no suitable method implemented for medium voltage, but there is for the low voltage topology. The corresponding description of this method is in the next paragraph. As soon as possible, this method will be adapted to the specific needs of medium voltage and integrated into its topology creation.

##### Low voltage level

 As in the case of medium voltage, there is almost no public information for the low voltage (lv) topology. This is due to the underground placement of the cables. However, the major advantage at the lv level is that there is some geographic information to derive the topology. The approach in DAVE uses the street and building information from OSM. By using geographic data as the basis for creating the low voltage topology, the only spatial constraint is that street and building information is available on OSM. Furthermore, substation information is additionally retrieved from the OEP (dataset: “ego_dp_mvlv_substation”^32^) and is queried to associate the name of the substation with a network node if it is located in its immediate vicinity. After collecting the necessary information, the first step in the generation process is to identify the house connection nodes by determining the centroid for all relevant building polygons. This includes residential, commercial, and industrial buildings. The second part of the node set is the network connection nodes, which by definition are located on a street. To obtain them, for each house connection node, an associated network connection node is searched by finding the minimum distance between the house and a street. The information about these node pairs can be used to create the initial network route type that connects the end users to the power network. In the next step of topology creation, the second type of network line is created. These lines connect the network connection nodes that are located on the same road. The street information is used to create the line routing by replicating the course of the street. To do this, an algorithm is iterated over all the roads in the network area under consideration. At each iteration step, the road direction is first checked against the longitude and reversed if necessary to obtain all road directions in a consistent style ascending the values of the longitude. Then, all suitable network nodes are searched based on the distance to the currently considered road and also sorted by their geographic longitude. The first node of the sorted set is used as the initial node. Based on the initial node, another sorting process follows, each searching for the nearest neighbour node starting from the last node found. This sorting method is important so that the later connecting lines follow the course of the road and not a zigzag-type pattern. When all nodes are in the correct order, connecting lines between adjacent nodes are created. During line creation, all coordinates of the road course that lie between the neighbouring nodes are filtered out. By adding these points, the resulting lines follow the course of the road. Finally, the line length is calculated using geographic information. To obtain a fully connected network, the last step of the low voltage topology is to link the interconnecting lines. This process also requires knowledge of road intersections. These intersections are determined by iterating over all roads and finding crossing points. Only the road intersections that are relevant to a connected network are added to the set of network nodes and used to adjust the line routes.

#### Power components

Based on the power network topologies, the DAVE dataset can be extended with information for transformers, power plants (renewable and conventional), and loads. The user has the possibility in the main function to define the components suitable to its individual use case. All of these elements are provided with information about the location and connection to an appropriate network node. The individual generation methods of the component types are described below.

##### Transformer

 The transformers are the link between the different voltage levels of the power network. The decision of which transformers to consider is based on the requested levels. They are important to obtain a coherent power network model when more than one voltage level is requested by the user. DAVE also generates the transformers at the transition to the higher and lower voltage levels that are not explicitly considered. This provides a better overview of the power transport to and from the network model under consideration. DAVE contains information for all types of different transformers, which are later included in separate tables in the resulting dataset. The basis of the information is the OEP (datasets: “ego_pf_hv_transformer”^33^, “ego_dp_hvmv_substation”^29^, “ego_dp_mvlv_substation”^32^ and “ego_pf_hv_bus”^30^). This currently results in a restriction to Germany for transformer generation. The OEP datasets contain different assumptions for transformer capacity because OSM’s baseline data does not provide this information^34^. The capacity for the ehv/ehv, ehv/hv, and hv/mv transformers were derived from the power capacities of the connected lines. Instead, the capacity assumption for the mv/lv transformers is based on load areas defined by a 360 × 360 meter grid^34^ and land use areas from OSM. Furthermore, the transformers are located in the centroids of the grid cells. The first step in the generation process of DAVE is the filtering of the transformers that are in operation and are additionally part of the considered area. Then, some parameter adjustments are made. The next step is to check if all necessary network nodes for the transformers are already available by the topology generation. In the case of mv/lv transformers, the appropriate low voltage node was searched from the set of road crossings based on the shortest distance. Missing nodes occur, for example, in the case of those transformers which connect from/to a voltage level that was not queried by the user. In case of missing nodes, they are added to the table of network nodes by searching the suitable nodes at the data for the not requested network levels. A peculiarity occurs when the user queries a low voltage network for an area where there is no medium/low voltage transformer. In this case, a transformer is created at the first low voltage network node. The external network representation is also connected to this transformer on the high voltage node side.

##### Energy production

 The power plants in DAVE are all components that feed energy into the power network. The associated generation process is divided into three sections. First, information is collected for power plants using renewable energy sources, and suitable connection points in the network topology are searched for. The second part is the same process for power plants based on conventional energy sources. Then, in the third part, interconnecting lines are created to connect the plants to the network. In the following, the process steps for plant creation are explained, which apply to both renewable and conventional energy sources. An overview of the energy sources considered is given in the Table 2.

**Table 2:** Power plant energy sources categorized in DAVE.

Type	Renewable	Conventional
Source	Biomass	Biomass
	Gas	Coal
	Geothermal	Gas
	Hydro	Gas_mine
	Solar	Lignite
	Wind	Multiple_non_renewable
		Oil
		Other_non_renewable
		Pumped_storage
		Reservoir
		Run_of_river
		Uranium
		Waste

The categorization is taken from the basic data retrieved from the OEP (datasets: “ego_renewable_powerplant”^35^ and “ego_conventional_powerplant”^36^). The process begins by importing the data and filtering based on the zip codes of the area specified by the user. Then some adjustments are made to the voltage parameter so that each power plant can be assigned to a voltage level. In case of missing information, it is assumed that plants with an installed capacity lower than 50 kW are connected to the low voltage network, otherwise to the medium voltage level. After all network level information is available, a second restriction is made by filtering power plants located within or below the considered network levels. If the user has queried a medium or low voltage network, a site improvement process is then performed for plants at that level. This step is necessary because these plants are assigned to an aggregate point in the OEP dataset and not to the origin coordinates. Fortunately, for most power plants, the address is provided so that the coordinates can be derived. For this purpose, the coordinates for each power plant are requested using the address information and “ESRI ArcGIS” as a geocoder. This process results in a power plant distribution with a higher level of detail. Subsequently, the power plants are divided according to their network level in preparation for the next process step, the distribution to the network nodes. This division is necessary so that each power plant can be connected to the most appropriate network node.

In addition, two other functionalities are necessary, which will now be described in more detail: the Voronoi decomposition and the power plant aggregation. In the context of power plants, the Voronoi function is used to divide the considered network area according to possible network nodes for connecting power plants. The next important function is used to aggregate a set of power plants. This is necessary to represent subordinate network levels that are connected to transformers of the considered network level. In DAVE, all power plants on the subordinate levels are always integrated into the dataset as well. In this process, the installed capacity of the plants connected to the same transformer is aggregated according to the energy source. The aggregation process results in one aggregated generator per energy source for each transformer. As described, there are functions to categorise the plants according to their network level, a voronoi decomposition of the area depending on the network nodes and the possibility to aggregate plants. On this basis, the distribution of power plants in the network can be made. There are two basic distribution cases that are used depending on the queried network levels. First, all power plants in the considered area are categorized according to their network level. Then, we iterate over these categories.

The first case is that the network level of the power plants should be considered in the iteration step according to the user input. Afterwards, the network plane is decomposed using the Voronoi function, with the network nodes of the plane as centroids. Each power plant is then connected directly to the network node of the Voronoi region in which it is located. The second case is that the network level of the power plants is lower than the lowest level required by the user. In this case, the transformers from the lowest user-requested voltage level to the voltage level below, are used as centroids for the Voronoi analysis. After that, the power plants are again intersected with the Voronoi regions. The difference with the first case is that the power plants are not directly connected to the corresponding transformer node. Instead, all power plants are aggregated according to their energy source and each is connected to the transformer node as a single power plant. After the renewable and conventional power plants have been generated, the final step is to create interconnection lines for the plants that are far from any existing network node. The boundary at which a power plant receives an additional line has been set at 50 m. If the plant is closer to the associated network node, it is assumed that the node belongs to the plant area. This assumption is made because the power plants are mostly given in point coordinates, but in reality they are an area. In this case, they will be directly connected to the network node. If the distance is greater, an additional node is created to match the power plant’s network level. The connection node is then changed to the newly created node in the plant parameters. A line is then created connecting the original node and the additional node. The line properties are taken from a neighbouring line located on the same network level and adjusted to the length of the new line. These additional lines and nodes can be recognized by the value “dave_internal” on the source parameter.

##### Energy consumption

 Energy consumption is the third possible component that can be generated for the power network. In DAVE, different methods are used depending on the voltage level and the type of consumption. For the network levels, the difference is that a more detailed consideration is required at the low voltage level than at the higher levels. Therefore, the load consideration on the low voltage level is building-specific and on the higher network levels, the loads are aggregated over regions. In addition, DAVE differentiates loads into residential, industrial, and commercial. The difference between the creation approaches of these load types lies in the input data. For residential loads at the low voltage level, building information and statistics on household sizes^37^ as well as average consumption per houshold size^38^ are used. The generation of residential loads at higher network levels and industrial and commercial loads at each network level follows an approach, based on land use information and assumed consumption factors. The factors are 2 MW/km^2^ for residential, 10 MW/km^2^ for industrial, and 3 MW/km^2^ for commercial^8^.

The different methods refer only to the generation of the active power portion of the loads. The reactive power is calculated for each load based on the active power generated and an assumed power factor. These power factors are derived for the different load types from information listed in the Table 3. The various methods for generating the active power loads are described in more detail below. Basically, loads in DAVE are always generated for the lowest network level that the user has queried and is connected to. For example, if the user requests a network model that includes the extra high and high voltage levels, aggregated loads will be generated and connected to the high/medium voltage transformers based on a Voronoi decomposition of the network area and transformer locations.

**Table 3:** Overview used power factors in DAVE.

Load type	cos($\varphi$) (researched)	cos($\varphi$) (used)
Residential	0.95 inductive (TU Darmstadt)^39^ 0.95 inductive (Uni Stuttgart)^40^	0.95 inductive
Industrial	0.70–0.82 (textile industry)^41^ 0.69–0.78 inductive (steel industry)^42^	0.75 inductive
Commercial	The research did not reveal any special power factor for this sector Therefore the same factor is assumed as for the industrial	0.75 inductive

**Figure 6:**
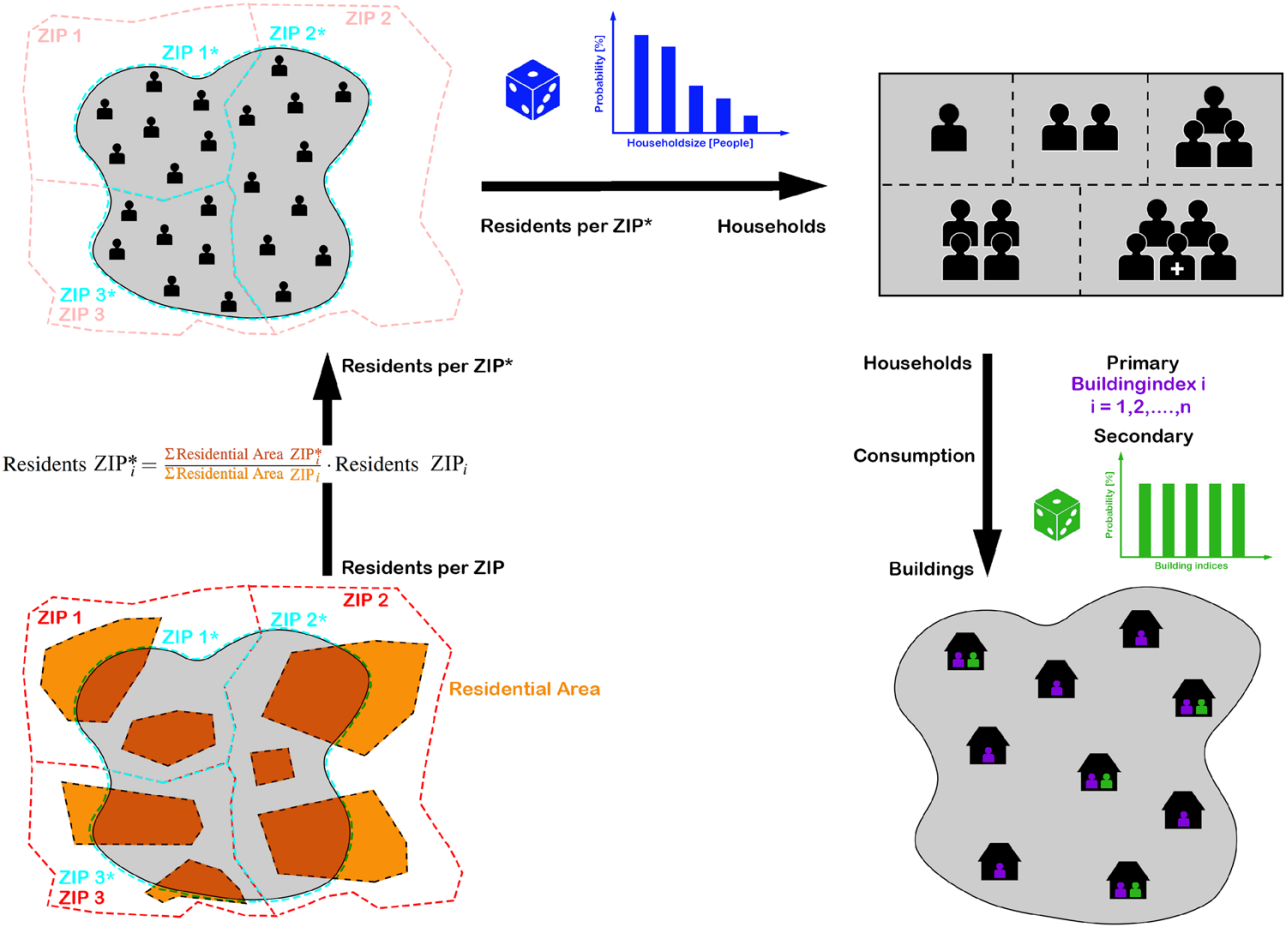
Generation process of the residential consumption at the low voltage network level.

The first case considered is load generation for the low voltage level. The first step in this process is the creation of residential consumption, which is the most complex and illustrated in Fig. 6. To begin, DAVE searches for possible load points by filtering out the building nodes from the low voltage topology that are labelled “residential”. Furthermore, it determines the appropriate state for the resulting buildings, as this information is needed in a later part of the load creation.

Another important parameter is the number of inhabitants in the considered area. For the predefined areas the population is already included, but if the user queries a dataset for a self-defined area, the population has to be calculated. The function behind the calculation takes the zip code areas (ZIP) and intersects them with the user’s own area. Then the resulting relevant parts of the zip code area (ZIP*) are intersected again, but this time with the residential areas. This results in the residential areas per ZIP* that are within the user-defined area. From this and from the total residential area in a ZIP, the percentage of the relevant residential area per ZIP* can be calculated for each ZIP. The resulting factors are then each multiplied by the total population of each ZIP to give the populations for the ZIP* areas.

The next step in this process is to distribute the loads using the population in the considered area. To do this, DAVE iterates over the ZIP* and first filters all relevant buildings located within the portions of the area under consideration. Using the previously assigned federal state, the associated average consumption per household size can be determined, which forms the basis for the loads. In addition, statistics for household size distribution per federal state is used. The load distribution is based on the population per area share. In doing so, the algorithm randomly distributes people as households among buildings using the household size distribution statistics as a weighting factor until the entire population is distributed. An implemented constraint is that each building first receives a minimum of one household. The rest of the population is then distributed completely randomly among the buildings, representing buildings with multiple dwellings. Finally, loads are created based on the allocated households and connected to the corresponding building node.

The second step in load formation for a low voltage network deals with the consumption for commercial and industrial buildings. For these types of loads, the land use information is used as a reference as well as the factors for average consumption per km^2^. The first step is to determine the total area in the region under consideration for each type. Multiplying this by the associated consumption factor yields the total consumption per load type. Then, iterating over the eligible buildings, an area percentage is calculated by dividing the building area by the total area of the land use type. This resulting area percentage multiplied by the total consumption for the load type gives the consumption for each building. Finally, each load is again included in the load table with the information for the corresponding building node.

The second case of load generation is when the low voltage level is not the lowest level queried by the user. This means that consumption must be generated for the medium, high, and extra high voltage levels. This process is based on the land use areas of the region under consideration and the average consumption factors per load type. Unlike the low voltage level, this approach also generates household consumption. The first step in this load generation process is to determine the lowest network level considered. Then, the nodes of the transformers connecting to the lower network level are used as centroids for a Voronoi decomposition. The resulting Voronoi regions are then intersected with the land use polygons to determine the land use areas associated with each Voronoi region. Based on this, an iteration is performed over all Voronoi areas or the transformers, calculating the aggregate area for residential, commercial, and industrial land uses. These aggregated areas are then multiplied by the appropriate land use factor for each Voronoi area. Finally, each transformer is assigned a load for each load type on the low voltage node based on the determined consumption.

#### Gas topology

The second energy sector that is partially available is gas. So far, the creation of the gas network topology is limited to the high pressure level. The data fundamentally used for the transport network come from the “SciGRID_gas” project (dataset: IGGIELGN^43^). These data include information for Europe. In addition to this information from the “GasLib”^22^ (dataset: GasLib-582 (version 2)^44^) was used to improve some assumptions in the SciGRID data. The GasLib dataset contains gas network element representations based on real network operator data. In the first processing step, the node and pipeline data from SciGRID are loaded from the DAVE database and reduced to the user-queried network area. Additionally, nodes outside the considered area are included to represent the gas exchange with neighbouring network areas. These nodes are identified by searching for pipelines that intersect the boundary of the considered area. Parameters are also added to determine whether a node can import or export gas. Both parameters are set to a boolean “False” by default, which means that the node is for interconnection only. In a later step of dataset creation, this can be changed during the creation of the gas network components. In the case of junctions connected to a source, the import value is set to “True”, and in case of connection to a sink, the export value changes. External nodes for gas exchange are defined as both import and export options. Then, during topology data creation, the pipelines are imported from the SciGRID dataset and also narrowed down to the area under consideration. Then, the roughness parameters of these data are improved with the values from the Gaslib dataset. To enable this, the Gaslib pipelines are clustered by length, and if there is more than one line with the same length, a median calculation is used to obtain the parameter values. Based on the length, the most similar line of the clustered GasLib lines is then searched for each SciGRID line and the roughness value is assigned. An example of a resulting high pressure network topology is shown in Fig. 5

#### Gas components

Equivalent to the power network, the gas network component is generated according to the gas topology. For this process step, the user has the possibility to select sink, source, and compressor as network elements in the main function. This is also based on the data from the “SciGRID_gas” project (dataset: IGGIELGN^43^). At this point, this is the only data source. After importing the information, it will be reduced to the user-defined network area. Also, some formatting is done and the DAVE-specific parameters are added. In the case of sinks and sources, the import and export parameters of the nodes they are connected to are adjusted.

#### Data cleanup

After all data generation processes are completed, additional functions are executed to clean the data. In this process, DAVE currently looks for topological errors rather than incorrect values. The cleanup process is performed for both the power system and gas system information. The procedure is the same, so the following process description applies to data from both sectors. To better check for topological gaps, a graph is first created from the information in the DAVE dataset. The set of all network nodes, independent of the network level, represents the nodes of the graph. Furthermore, the set of all lines and transformers forms the edges of the graph. The next step is to cleanup the disconnected elements. For this, an algorithm finds out which nodes are connected to each other. This results in the knowledge of disconnected parts of the graph. Then, the size of the respective parts is determined based on the contained nodes. It is assumed that it is a data error if a subgraph is formed from less than four nodes. The set of nodes from all detected erroneous subgraphs forms the basis for determining the unconnected elements. Then, all network lines and components are checked to see if they are connected to any of these nodes. Finally, in this cleanup step, all nodes, lines, and components that are part of a subgraph are deleted from the DAVE dataset. The next step in the cleanup process deals with incorrectly connected lines. This involves checking whether the start and end points of a line are identical and therefore connected to themselves. In this case, the found lines are removed from the DAVE dataset. After the data cleanup the main process of DAVE and thus also the dataset to be generated is finished. The resulting dataset is passed to the user and optional processes still follow depending on user settings.

#### Plotting

**Figure 7:**
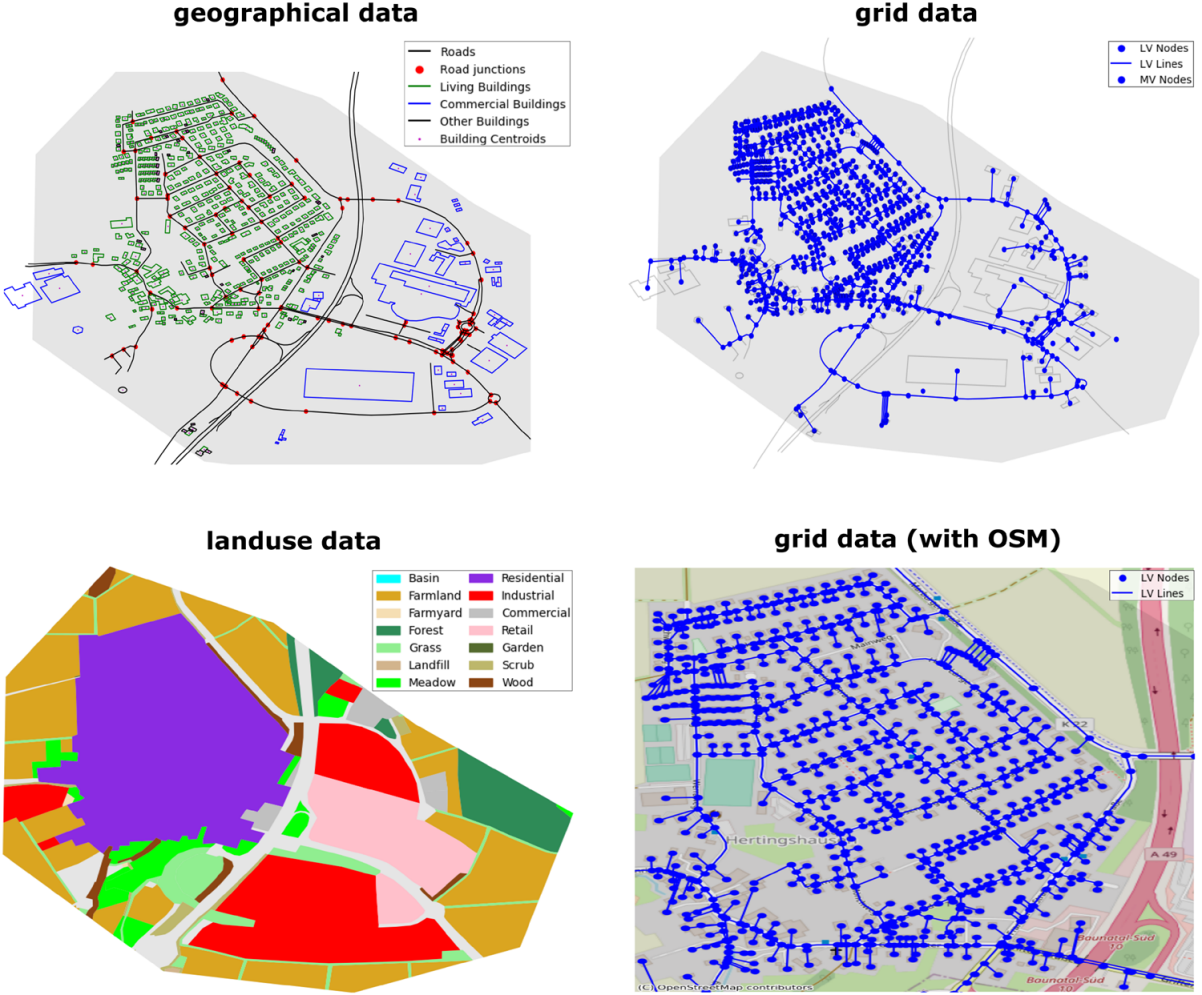
Plotting options in DAVE.

One of the optional processes is plotting the information from the DAVE dataset. This provides the user with a visualized overview of the data with geographic mapping. In this option, DAVE creates different types of plots, all of which have a background layer as a base, which greyed out the network area polygon. Currently, four different plot types are implemented that are visualized in Fig. 7.

- *geographical data:* Overview of the geographical data, independent of the generated energy network topologies.
- *grid data:* Presentation of the resulting network topology data. Optionally, an OSM map can be displayed in the background.
- *landuse data:* Illustration of the different landuse areas within the considered region.

#### Output creation

The final step of the data creation process is the optional conversion to formats for further simulations. The user has the option of specifying several desired conversion formats through the main function. Conversion gives DAVE more flexibility and ease of use by providing the user with the individual data set in the format of the preferred simulation tool. Currently, converters for pandapower, pandapipes, gaslib and MYNTS are implemented.

*pandapower*^45^ is a Python-based open-source power system modeling, analysis, and optimization tool licensed under the 3-clause BSD license. The basis for this tool is the combination of the data analysis library pandas and the power flow solver PYPOWER. Within the converter, DAVE uses several pandapower-in-build functions for further error cleanup and optimization on the converted dataset. Furthermore, there is the opportunity to convert from pandapower format to PYPOWER, MATPOWER, PowerFactory and CIM CGMES using the pandapower tool.

*pandapipes*^46^ is a Python-based open-source tool for modeling, analysis, and optimization of fluid systems licensed under the 3-clause BSD license. The basis for this tool is the data analysis library pandas.In combination with pandapower, the tool provides the possibility of multi-energy network analysis. Furthermore, the tool gives the opportunity to convert from pandapipes format to Stanet.

*GasLib*^22^ is an open available library of gas network instances. With the DAVE included converter, the user has the opportunity to get the resulting dataset in a format (xml) compared to the gaslib one.

*MYNTS*^47^ is a closed source mulitphysical network simulator. The software gives the opportunity to simulate gas transport, water supply and electrical power networks.

The raw dataset of DAVE will be saved as well. Therefore the data will convert into a user-chosen format. At this time there are JSON, HDF5 and geopackage as possible output formats available for selection.

### Outlook

The ultimate goal of DAVE is to cover as many different network model requirements as possible. As seen in the previous chapters, DAVE already covers many different areas in the context of energy network modeling and can provide a wide variety of open data. However, there are also many topics that have not yet been covered. This outlook is intended to provide an overview of some potential enhancements that could be implemented in the future.

Generally, the existing data should be further improved and more data sources should be added. Specifically for geospatial data, building information needs to be expanded for more detailed viewing. Also, the viewing area should be enlarged for each data category, since most of the data is limited to Germany so far. Furthermore, on the data side, the fusion algorithms can be improved and extended. To make the use and access to the data more user-friendly, they should be adapted to the FAIR criteria. In addition, it is planned to implement a data quality metric to evaluate the usefulness of the input data or the data resulting from DAVE. A very useful new feature would also be the ability to pass your own data to DAVE and include it in the generation process.

In the area of network topologies and components, there are plans to extend DAVE to include gas distribution networks, thermal networks, ICT networks and DC systems in the electricity sector. In addition, DAVE currently generates a coherent network topology in each sector. A more realistic way would be to divide the generated structures into network groups. Suitable algorithms, possibly based on clustering methods, would have to be developed for this purpose. Another expansion step would be to implement time profiles for energy generation and consumption to enable dynamic simulations with the resulting network models. Scenario data could also be integrated into DAVE. For this purpose, suitable algorithms have to be developed to regionalize the scenario data to fit the network nodes. The first development steps have already been taken for this extension task.

The performance of the tool itself could be optimized by parallelizing the processes. Furthermore, the software-as-a-service platform with all its components should be improved to make it more stable and robust against many user requests.

## Data records

This section gives an overview of all datasets used in DAVE. The philosophy is to use the raw data as input. This makes it easier, for example, to replace it when the data is updated, provided that the format remains the same. Necessary adjustments are made as part of the generation process and only when needed. In general, the emphasis is on the use of open data so that DAVE is widely usable and transparent. In addition, the number of data sources is continuously expanding. Table 4 provides an overview of all input datasets currently in use for each resulting data category, and the following subsections describe these input datasets in detail.

**Table 4:** Overview about currently used input data for each resulting data category.

Data category	Type	Input data	Resolution
Network area	Postal codes/town names	^48^	Germany
	Federal states	^49,50^	Germany
	NUTS regions	^51^	Europe
	Shapefile/polygon	User defined	World
Geographical data	Roads	^24^	World
	Buildings	^24^	World
	Landuse	^24^	World
	Waterways	^24^	World
	Railways	^24^	World
Power topology	Extra high voltage	^28,30,31^	Germany
	High voltage	^28–31^	Germany
	Medium voltage	^29,32^	Germany
	Low voltage	^24,32^	Germany
Power components	Transformers	^29,30,32,33^	Germany
	Renewable power plants	^35^	Germany
	Conventional power plants	^36^	Germany
	Loads	^24,37,38^	Germany
Gas topology	High pressure	^43,44^	Europe
Gas components	Sinks	^43^	Europe
	Sources	^43^	Europe
	Compressors	^43^	Europe

### Geographical data

The main source of geographic data is OpenStreetMap^24^. Instead of closed datasets, there is a database where information for a specific geographic area can be queried. In DAVE, different information is needed in different steps of the process. Table 1 gives an overview of the tags that are used when needed. OSM provides a worldwide overview of geographic information based on the “Open Data Commons Open Database License 1.0”^52^ license.

The dataset for postal code areas is based on information from the “suche-postleitzahl.org”^48^ website and provides data for the five-digit postal code areas in Germany. The data contains both the geographic description and the associated name, which is also used in DAVE to define areas based on the input of city names. In addition, the dataset for DAVE has been extended to include the population of the respective region. This information is based on the 2011 Census, which was published by the Federal Statistical Office in 2015 and is also available on the website “suche-postleitzahl.org”. In addition, some data gaps were filled, areas with the same postal code were reduced to a geometric object, and the area for each region was calculated. The individual datasets used are in csv and xlsx formats and are subject to the “Open Data Commons Open Database License 1.0”^52^ license.

The dataset of federal states is based on information from the website “arcgis.com” and contains data on administrative boundaries in Germany. These data originally come from the Federal Agency for Cartography and Geodesy and are licensed “dl-en/by-2-0”^53^. For DAVE, the dataset was extended by the population figures of the individual federal states. The population data come from the dataset “Population: federal states, reference date (12411-0010)”^50^ from the Federal Statistical Office database, resulting from the 2011 census and subject to the “dl-de/by-2-0”^53^ license. In addition, the geographic data for the state of Baden-Württemberg were adjusted because a small area of Germany was outside the boundaries in the source data, resulting in the substation of “Schwörstadt” not being part of the state region.

NUTS regions are available for different years (2013, 2016, 2021) in DAVE and include all three levels. This information is provided by the Statistical Office of the European Union (Eurostat) through the datasets “NUTS_RG_01M_2013_4326”^51^, “NUTS_RG_01M_2016_4326”^51^ and “NUTS_RG_01M_2021_4326”^51^. The data can be downloaded in various formats and are subject to the “Creative Commons Attribution 4.0 International”^54^ license.

### Electrical data

The dataset “ego_dp_ehv_substation”^28^ of the Open Energy Platform provides information on substations in Germany. It contains data on substations connecting the extra-high voltage level to itself and also on those connecting the extra-high and high-voltage levels. The data is available both in csv format and via api and is subject to the “Open Data Commons Open Database License 1.0”^52^ license.

The “ego_dp_hvmv_substation”^29^ dataset of the Open Energy Platform provides information on substations in Germany. It contains data on substations connecting the high and medium voltage levels. The data is available both in csv format and via api and is subject to the “Open Data Commons Open Database License 1.0”^52^ license.

The “ego_dp_mvlv_substation”^32^ dataset of the Open Energy Platform provides information on substations in Germany. It includes data on substations connecting the medium-voltage and low-voltage levels. The data is available in both csv format and via api and is subject to the “Open Data Commons Open Database License 1.0”^52^ license.

The “ego_pf_hv_transformer”^33^ dataset of the Open Energy Platform provides information on transformers in Germany. It contains data on transformers connecting the extra-high voltage level to themselves and also on those connecting the extra-high and high-voltage levels. The data is available both in csv format and via api and is subject to the “Open Data Commons Open Database License 1.0”^52^ license.

The “ego_pf_hv_line”^31^ dataset of the Open Energy Platform provides information on lines in Germany. It contains data on lines at extra-high and high-voltage levels. The data is available both in csv format and via api and is subject to the “Open Data Commons Open Database License 1.0”^52^ license.

The “ego_pf_hv_bus”^30^ dataset of the Open Energy Platform provides information on nodes in Germany. It contains data on nodes at the extra-high and high-voltage levels. The data is available both in csv format and via api and is subject to the “Open Data Commons Open Database License 1.0”^52^ license.

The “ego_renewable_powerplant”^35^ dataset of the Open Energy Platform provides information on renewable power plants in Germany. It contains data on renewable energy power plants at all voltage levels. The data is available both in csv format and via api and is subject to the “Open Data Commons Open Database License 1.0”^52^ license.

The “ego_conventional_powerplant”^36^ dataset of the Open Energy Platform provides information on conventional power plants in Germany. It contains data on conventional power plants at all voltage levels. The data is available both in csv format and via api and is subject to the “Open Data Commons Open Database License 1.0”^52^ license.

### Gas data

The “iggielgn”^43^ dataset of the SciGRID_gas project provides information about the gas transmission network in Europe. It contains data on nodes, pipelines, compressors, consumers, LNG, production, and storage. The data is available in csv and geojson formats and is licensed under “Creative Commons Attribution 4.0 International”^54^.

The dataset “GasLib-582 (Version2)”^44^ from the GasLib library of gas network instances provides representative information for the gas transmission network in Germany. It contains data on nodes, pipelines, sinks, sources, compressor stations, valves and resistors. The data is available in xml format and is subject to the “Creative Commons Attribution 3.0 Unported”^55^ license.

### Demographic data

The household size data are based on the “Households by Household Size - Regional Level (12111-31-01-4-B)”^37^ dataset from the “Census 2011” and provide information on the number of households by size for each federal state in Germany. The original dataset is available from the Regional Database Germany^56^ and is subject to the “Data License Germany - Attribution - Version 2.0.”^53^. For usage in DAVE, household types were reduced to 1 to 5+ person households. In addition, the percentages of household sizes by state were added to the dataset.

The average household consumption data is based on the “Stromspiegel für Deutschland 2019”^38^ and provides information on the average consumption per household size for each federal state in Germany. In addition to persons per household, the original dataset is subdivided into apartments and houses, and with and without water heating by electricity. To obtain average values for each household size, the dataset was first cleaned by calculating the average of the interval from the middle category D. The average was then calculated from the average of the four categories. Then, the average was calculated across the four categories to obtain a value for each household size.

## Technical validation

So far, two different validations have been performed to compare the performance of DAVE against network models from other sources. The first validation is related to the low voltage level for a district of the city of Bamberg. In this comparison, the data resulting from DAVE is compared to real information from the corresponding distribution system operator (DSO) “Stadtwerke Bamberg”^57^. Second, a dataset generated from DAVE for the extra high voltage level within Germany is compared with the corresponding data from SimBench^18^. In the following, the results of both comparisons are presented in detail.

**Figure 8:**
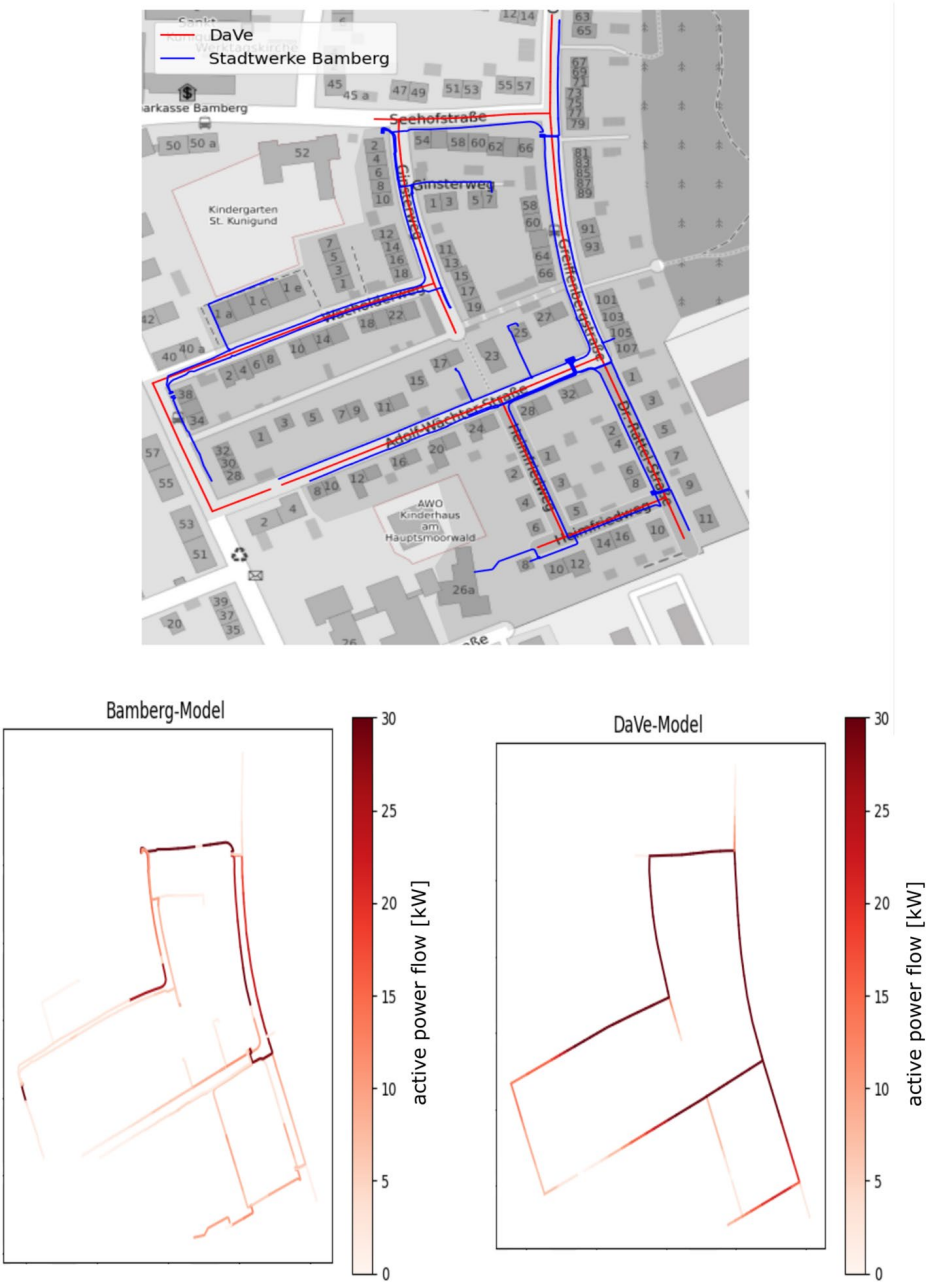
Comparison between DAVE and Stadtwerke Bamberg for a low voltage network. Top: Comparison of the lines routes. Bottom: Comparison of the lines active power flow [kW].

### Low voltage validation with Stadtwerke Bamberg

As mentioned above, data from the network operator “Stadtwerke Bamberg” were available for the comparison of the low voltage method. The provided dataset represents a district of Bamberg and contains information about the network topology and the main network elements. To comply with confidentiality, customer-specific data such as house connection lines were not part of this study. In the first step, the network topologies were compared graphically. Figure 8 shows in the upper area the resulting lines from DAVE in red and the line routing of the DSO’s model in blue. In this visualization, it can be seen that the cable routes basically match well. In reality, however, cables are laid on both sides of the road to keep civil engineering costs low. This circumstance has not been taken into account in DAVE so far. Another difference that stands out is some additional cables in the network operator model. This can have two different reasons. The first reason can be cables that run along rows of houses. In DAVE there is no rule implemented so far when a house is part of a row. Therefore all houses have their own connecting line. The second reason can be missing street information in DAVE. In the base dataset of OSM sometimes small streets are tagged with the wrong tag and due to this they are filtered out during the generation process. Adding these tags would not be a solution, because so much wrong road information is included, which leads to further topology errors. The next step was to compare the network elements, resulting in Table 5. For reasons of data protection, no network node-specific analysis was carried out, but aggregated values for the area were considered. Based on the values, it can be seen that the number of lines matches well, but the line length in the network operator data is twice as high. This is partly due to the fact that the lines in DAVE are partly divided into line sections, and partly due to the fact that the network operator’s plant is twice as long. The number of generators is the same in both models and also the installed active power has only a small deviation. On the load side, however, there is a larger difference in both the quantity and power values. This overestimation results from load generation based on population and the current lack of building information for the height and number of dwellings within. For example, assuming an even distribution of population across residential areas results in an overestimation in areas with many single-family homes and an underestimation in regions with high-rise buildings that contain many households.

In addition, DAVE generates time-static loads based on average consumption values per household that deviate from a specifically considered time step. In this comparison, the measured consumption values were taken from a Wednesday at 8 pm on the network operator side. Finally, it is noticeable in the values that in both datasets there is a substation for the medium voltage level and they are also in the same location. In the third and final part of the comparison, the active power flows of the lines were compared. The active power flow results are visualized in Fig. 8 below for both network models. In this figure, the colour scale represents the amount of active power, low values are represented by a white colour and high values are displayed in red. It can be seen that the networks have similar characteristics in their power flow. In the DAVE model, the lines basically transport more power, which leads to a generally darker color. This is due to the overestimated household loads, which draw more power from the local transformer than in the network operator model. In summary, DAVE generally provides comparable results in the same order of magnitude for the low voltage level. The topology generated by DAVE is similar to that of the network operator. More operator and expert knowledge need to be integrated into the generation process to account for special topology features such as terraced houses. Furthermore, the comparison showed that the estimation of loads needs to be improved.

**Table 5:** Comparison of the elements between DAVE and Stadtwerke Bamberg for a low voltage network.

Network element	Stadtwerke Bamberg	DAVE
Lines	147	144
Circuit length [km]	2.43	1.26
Generators	4	4
Generators active power [kW]	21.47	21.51
Loads	244	835
Loads active power [kW]	115.38	287.73
Loads reactive power [kVar]	38.00	94.57
mv/lv-Transformers	1	1

### Extra high voltage validation with SimBench

To validate DAVE’s extra-high voltage level generation process, a network model was created for the whole of Germany and compared with a suitable model from the SimBench project. The SimBench project created freely available benchmark power network datasets for each voltage level. This sounds very similar to the work done by DAVE, but there are some differences between these two tools. In SimBench, there are 13 predefined basic network models that can be combined. In contrast, in DAVE there is the possibility to create an individual network model by defining the network area completely free. In addition, DAVE allows the user to request a model that includes all voltage levels, whereas in SimBench only two levels can be combined automatically. Moreover, in SimBench only the maximum and high-voltage have a relation to reality. An advantage of SimBench compared to DAVE is that all network models have been manually tuned and verified by experts. In addition, the generators and loads in the SimBench models have time series that are not yet available in DAVE.

**Figure 9:**
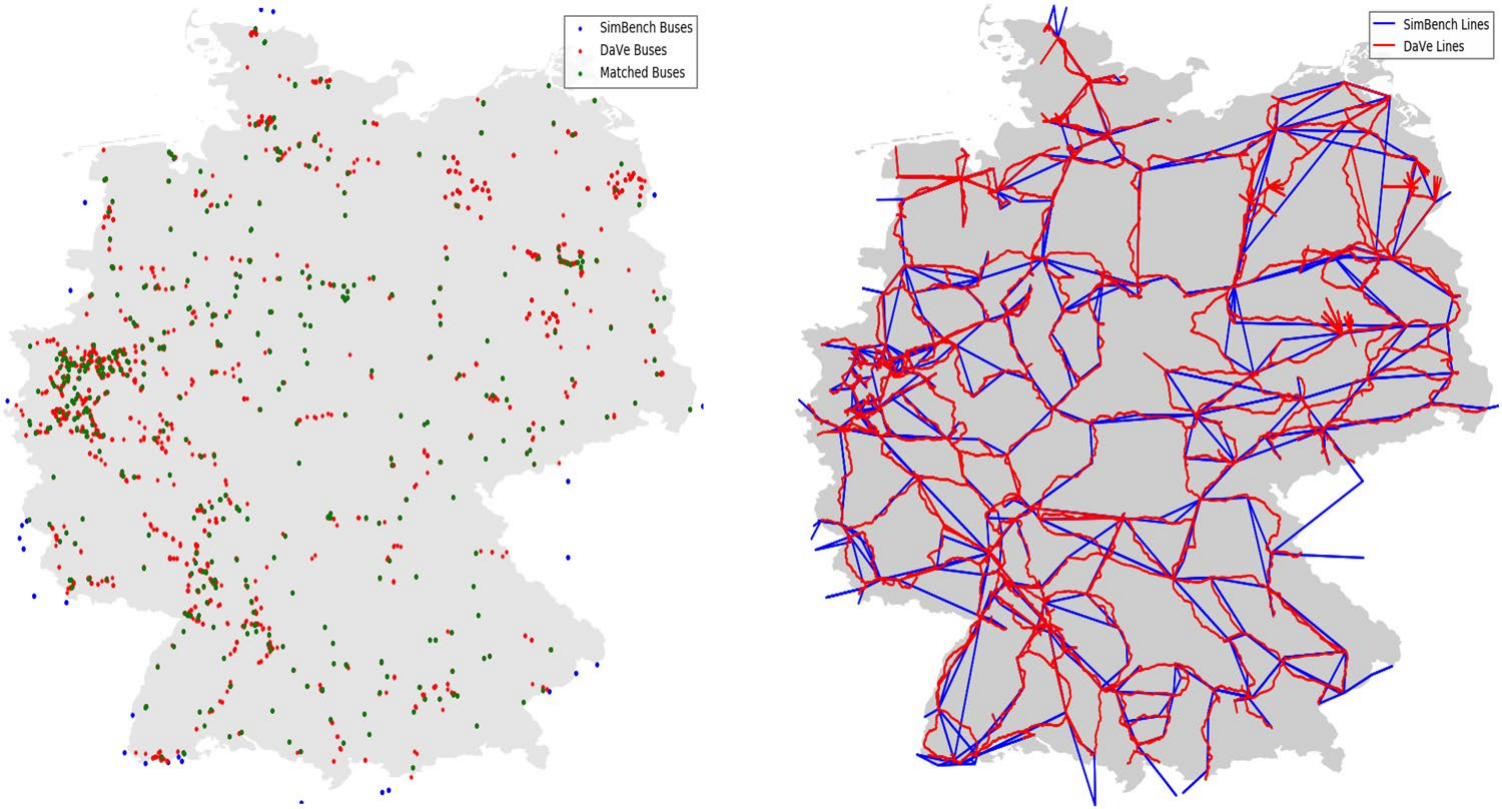
Comparison of the topology between DAVE and SimBench for an extra high voltage network.

For the comparison of the extra high voltage level, the network with the SimBench code “1-EHV-mixed–0-no_sw” was used. Both, this network model and the DAVE one are based on the data from OSM. They differ in the data processing performed in SimBench by the project team, whereas DAVE uses preprocessed data from the OEP instead. In the first part, the topology of the two models was visualized and compared against each other. The result graphics are separated by nodes and lines and shown in Fig. 9. This gives an overview of the model structures and shows basic differences and similarities in the geographic node locations and line trajectories.

At first glance, they look very similar, yet there are some differences to be noted. In the node comparison, each node was categorized and colored according to which dataset it is included in. If the nodes are included in both the SimBench model and the DAVE model, they were colored green. These nodes are distributed over the entire network area and at the points where the SimBench and DAVE lines cross. All SimBench nodes in the interior of the area were counted to this node category after verification. From this result, it can be concluded that the locations of the substations match very well. The second node category are those that are only included in the SimBench dataset. These nodes are colored blue and are located at the edges of the network areas. This effect is due to the fact that SimBench also maps the interconnectors to neighboring countries. In DAVE these nodes are missing because DAVE only generates data for the defined area, which in this case was Germany. The last category is the red-colored nodes, which represent only a part of the DAVE network model. These additional nodes compared to the SimBench model can be explained by looking at the line comparison graph.

Both models have a similar line structure, but DAVE also represents the real geographic line routes instead of SimBench’s direct node-to-node connections. This advantage of the DAVE model results in additional nodes as well as more lines since each line is divided into multiple segments. A second reason for the additional nodes and lines is that generators in DAVE receive an interconnection line if the generator is more than 50 meters away from its network connection node.

After the graphical check of the two network models against each other, the second part of the comparison was to take a look at the number of network elements. A listing of the included components can be found in the Table 6. First, the difference in the number of buses and lines can be seen, which has already been explained in the graphical comparison. The described more detailed line paths in DAVE are the reason why the circuit length in DAVE is higher.

A major difference is the number of external networks resulting from the different approaches representing power control. In the SimBench model, all nuclear power plants were represented by an external network. In contrast, DAVE does not use external networks at the extra-high voltage level because external networks look like superimposed networks, which could be confusing to the user. Instead, the rotating generator with the highest power is defined as the slack bus. Using only nuclear power plants as a slack bus is not practical in DAVE because the user could define a network area that does not include this type of power plant.

In addition, the number of rotating and static generators differs. This results from the fact that in DAVE the generators from the underlying network levels are considered in aggregated form and connected to the corresponding transformers. In addition, each aggregated generator is divided according to its energy source. SimBench, on the other hand, only considers power plants with an operating voltage of 110 kV or higher. This approach by DAVE also leads to a higher assumption of installed power for rotating and static generators, which can be seen in Table 7.

The next power components in this comparison are loads. Based on Table 6, there are also large differences between the set of elements in the two models in the consumption sector. Nevertheless, Table 7 shows that the assumption of installed active power is very similar. Since the load capacity in SimBench is derived from ENTSO-E information, it means that the load estimation in DAVE is quite good for the extra-high voltage level. On the other hand, the reactive power is overestimated and should be improved.

The last network element in this comparison are the transformers. In SimBench there are almost exactly twice the number of ehv/ehv transformers. This difference results from the approach of redundant transformers instead of single ones in DAVE. The reverse is true for the ehv/hv transformers. In DAVE, there are significantly more, because all underlying high-voltage networks including the associated substations are taken into account there. In SimBench, on the other hand, a maximum of two high-voltage networks are available that are connected to the extra-high voltage level, so that a large number of substations are neglected.

**Table 6:** Comparison of the elements between DAVE and SimBench for an extra high voltage network.

Network element	SimBench	DAVE
Buses	571	3775
Lines	849	5704
Circuit length [km]	32,426	48,896
External networks	7	0
Rotating generators	338	528
Static generators	225	4900
Loads	390	1218
ehv/ehv-Transformers	209	103
ehv/hv-Transformers	9	406

**Table 7:** Comparison of the installed power and the power flow results between DAVE and SimBench for an extra high voltage network.

Parameter	SimBench		DAVE	
	Installed	Power flow	Installed	Power flow
External networks active power [MW]	9516	− 11,335	–	–
External networks reactive power [Mvar]	3128	1056	–	–
Rotating generators active power [MW]	73,095	73,095	90,962	8416
Static generators active power [MW]	12,842	12,842	88,688	31,085
Loads active power [MW]	72,145	72,145	73,798	39,486
Loads reactive power [Mvar]	23,780	23,780	43,032	20,448
Minimum node voltage [p.u.]	0.77		0.95	
Maximum node voltage [p.u.]	1.09		1.03	
Maximum line loading [%]	512		38	
Maximum transformer loading [%]	151		27	

Finally, some power flow results were contrasted, as can be seen in Table 7. During the Comparison the AC power flow with pandapower did not converge for the DAVE model. This result is an important finding for further development. Nevertheless, in order to make a comparison, the DAVE network model was optimized to converge using a DAVE built-in optimizing function based on the optimal power flow (OPF) of pandapower. This algorithm searches under the use of the OPF on optimal load and generator constellation where the load flow converges. The degrees of freedom are the active and reactive power of the loads, static generators and rotating generators which can be set between zero and the installed capacity. Furthermore, the optimization is restricted by default comply a node voltage between 0.95 p.u. and 1.05 p.u. as well as a maximum line and transformer loading of 100 percent.

The power flow results are very different between the two models, which can be attributed to two issues. The first issue is the power of the network elements. In the SimBench model, all elements are fully operated at their installed capacities. In the DAVE network model, on the other hand, the installed capacities of loads and generators have been drastically reduced due to optimization. This different handling of installed capacities also leads to the second aspect of the difference, compliance with operating limits. In SimBench, there are unrealistic values for the minimum and maximum node voltage. In addition, the maximum load on the lines and transformers is too high. In the results for the DAVE model, the operating limits are respected, which is because this is the main criterion in the optimization function of DAVE.

The conclusion from this comparison is that it is not possible to deduce which network model is better and that this must be decided based on the use case. In addition, important insights for further development were gained through the validation.

## Usage notes

Based on DAVE’s philosophy of providing an easy way to obtain a dataset or network model for an individual area, it will also be easy to use. Therefore, the DAVE team has decided to make the tool available through a software-as-a-service platform, rather than installing it on the user’s own computer. Currently, this platform is still under construction and is entering a beta testing phase. In this test phase, new users cannot yet register themselves. Interested parties must contact one of the authors to get a free account. In the future, when the test phase is completed, it is planned to provide the possibility to create your own account directly on the DAVE website.

The software-as-a-service platform consists of several parts to provide different ways of interacting with DAVE depending on the users’ needs. There is a graphical user interface (GUI), an application programming interface (API), and a client that makes using the API more convenient. In addition, a separate database runs in the background, primarily providing DAVE with the information it needs and secondarily providing the raw data for users. For performance reasons, data from external databases is also integrated into the own database and kept up to date with suitable algorithms. A second background process of the platform is authentication. To ensure that DAVE is not available on the Internet completely unprotected, it was decided to secure the GUI and API functions with upstream user authentication. A DAVE account is free of charge and can currently be requested from the authors. For more information about the tool and using the software as a service, please visit the DAVE website at http://databutler.energy/.

### Graphical user interface

**Figure 10:**
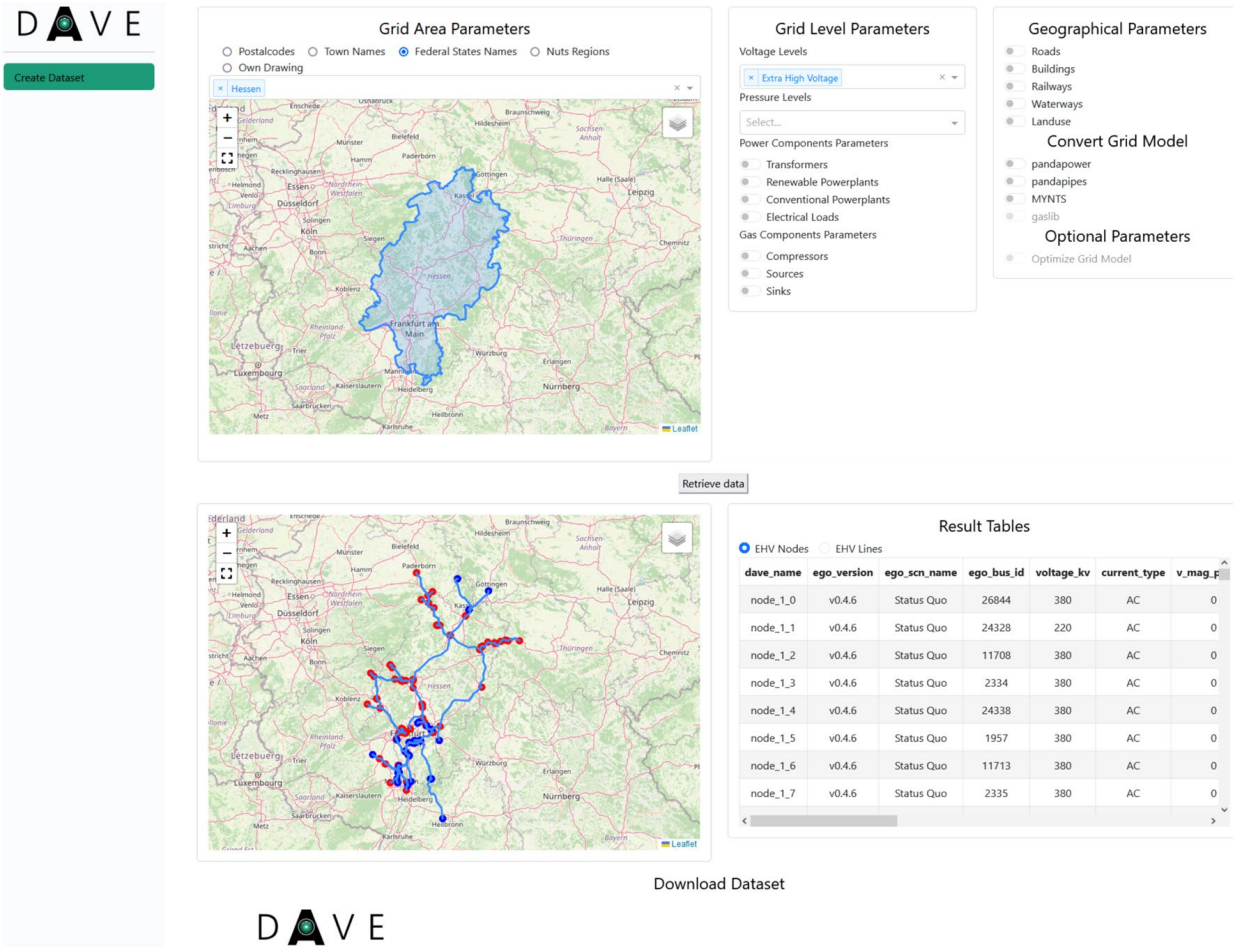
Look of the first version of the graphical user interface.

 The graphical user interface of DAVE is an easy way to get an individual dataset from the tool. It can be used directly from the browser and therefore no programming knowledge or additional software is required. When you visit the GUI, the login dialogue opens first, where you authenticate yourself with your credentials. After successful login, you enter the GUI and can use the dropdown menus as well as the buttons to set up your individual data query. A special feature of the graphical interface is the possibility to draw your area of interest on a map in the browser. This makes it very easy to define an area according to your needs. After everything is set up, the generation process of DAVE can be started by clicking on the retrieve-button. Depending on the size of the area under consideration and the type of data to be queried, this can currently take anywhere from a few minutes to hours. Then the results are visualized on a map as well as displayed in tables. Additionally, there is the possibility to download the resulting data and open it e.g. with the DAVE client. The current appearance of the first GUI version is shown in Fig. 10.

### Application programming interface

 DAVE’s application programming interface provides the ability to retrieve a custom dataset from the tool directly in your favorite Python development environment. First, it is important to log in here as well. To do this, you can send your credentials to the API and receive back a token that will be used for authentication in subsequent API requests. More detailed instructions on how to use the API can be found on the DAVE website. The API provides several options for retrieving data. The main function is to request a data set from DAVE. You can send the parameters to define the dataset and the request will start the generation process. After the process is finished, the results are returned as a string in DAVE format, which should be converted to a dictionary for better use. In addition to this function, there is the possibility to query raw data from DAVE’s own database. Functions are available to query an overview of all data stored in the database, to obtain a table from the database, and to obtain a table filtered by geography. The resulting data is returned as pandas/geopandas tables and converted to a string.

### Client

 The DAVE client is a small add-on tool that simplifies the handling of the API and needs to be installed locally. It is also still in the testing phase, so if you are interested in using it, you need to contact the authors. Soon it will provided via pip. It contains the same functionality as the API, but the user does not have to deal with the processes of authentication, request, and conversion. In addition to these functions, the client also provides the ability to read DAVE-generated records and convert them into other data formats.

## Data Availability

The data sources for all data used by the featured tool are included in this published article. An overview is given in the “Data records” section. The resulting data are individual and depend on the user’s requirements. A description of how to use the tool can be found in the “Usage notes” section of this article.

## Code availability

The DAVE source code is licensed under a 3-clause BSD license and currently available in the version 1.2.0. Additionally, the code is not openly available because the general usage of DAVE should be done via the previously described software as a service platform. With this platform and associated components, users can benefit from all DAVE functionalities. In case you want to contribute the DAVE development feel free to contact the authors.
